# Time is crucial in malignant tumor cases: Speeding up the process of patient-specific implant creation

**DOI:** 10.3389/fonc.2022.904343

**Published:** 2022-09-21

**Authors:** Simon Spalthoff, Narin Nejati-Rad, Björn Rahlf, Philipp Jehn, Nils-Claudius Gellrich, Fritjof Lentge, Philippe Korn

**Affiliations:** Department of Oral and Maxillofacial Surgery, Hannover Medical School, Hannover, Germany

**Keywords:** head and neck squamous cell carcinoma, patient-specific implant, time-to-treatment, mandible, workflow, computer-aided design, artificial intelligence

## Abstract

**Purpose:**

Patient-specific implants are commonly used to reconstruct lower jaw defects following surgical treatment for head and neck squamous cell carcinoma. The planning process of surgery is time-consuming and can delay the “time to surgery,” which should be as short as possible. Therefore, this study aimed to evaluate the planning process to speed up and identify any sources of problems.

**Patients and methods:**

In this retrospective study, we enrolled patients who underwent continuous resection of the mandible in combination with reconstruction with a patient-specific implant between 2016 and 2021. The predictor variables were in-house training of the engineers and implant complexity (complex [with additional features] vs. less complex [resembling standard reconstruction plates]). The outcome variables were the duration of communication, message length, and the need for synchronous communication or modifications to the original design. Descriptive and univariate statistics were computed, and statistical significance was set at P < 0.05.

**Results:**

The data from 83 patients were included in this study. The mean duration of communication was 14.05 ± 13.58 days. The implant complexity and training status of the engineer had no statistically significant influence on the primary outcome variables. As for the secondary outcome variables, the implant complexity significantly influenced the chance that the planned operation had to be postponed (15/16 [93.75%] were complex cases, P = 0.001). The most frequent cause of problems in the planning process was an insufficient dataset, which was not dependent on the type of imaging.

**Conclusions:**

The overall duration of the patient-specific implant creation process is too long to meet oncological requirements. Therefore, standardization of the planning process to accelerate implant creation is of utmost importance. In addition, a common standard imaging format (independent of the type of imaging) for oncological cases could eliminate all delays caused by insufficient datasets in the future.

## Introduction

Head and neck squamous cell carcinoma (HNSCC) is the sixth most common cancer worldwide, with an increasing incidence per year. It accounts for approximately 3% of new cancer diagnoses in the United States and almost 900,000 new cases annually worldwide, resulting in approximately 450,000 deaths worldwide in 2018. HNSCC, the most common head and neck cancer accounts for more than 90% of all cases, often arises from the epithelium of the oral cavity, oropharynx, nasopharynx, hypopharynx, and larynx (1–4).

Treatment for HNSCC usually involves a diagnostic and staging phase followed by treatment *via* a selection or combination of surgery, radiotherapy, or chemotherapy (4). An important prognostic factor is the time between the initial diagnosis and the start of treatment (time-to-treatment initiation) (5). An increase in time-to-treatment initiation seems to be associated with worsening mortality, even if this relationship may be multifaceted, with sociodemographic issues, management of comorbid conditions, and complexity of treatment modalities contributing to increased time-to-treatment initiation and decreased overall survival (6–8).

Time to surgery (TTS) is a crucial factor in the surgical treatment of HNSCC. A study by Rygalski et al. in 2020 showed a 29% increase in mortality for certain tumor locations when oropharyngeal surgery was delayed by more than 30 days relative to surgery performed within 30 days. Additionally, the patients who had a TTS longer than 67 days were independently predicted to experience worse overall survival than those with a TTS of 67 days or less. Rygalski et al. concluded that reasonable efforts should be made to expedite primary surgery for HNSCC, especially in the oropharynx and oral cavity subsites (9).

This commonly known relationship between time, tumor progression, and tumor survival has led to a recommendation for HNSCC treatment by Lauritzen et al. in collaboration with the Danish Head and Neck Cancer Group: 21 calendar days for diagnosis; 7 or 11 days for the planning of surgery or radiotherapy, respectively, and therefore, a total of 28 or 32 calendar days from suspicion of cancer to initiation of surgery or radiotherapy (10).

The planning of surgery, which should be performed within 7 days, includes aspects of patient-specific tumor therapy or patient-specific reconstruction of tumor therapy-induced hard and soft tissue defects. HNSCC of the alveolar crest or mouth floor, for example, can lead to partial resection of the mandible. State of the art therapy of mandibular defects nowadays includes the use of patient-specific implants to reconstruct the mandible, with or without bone grafts (11, 12). Patient-specific implants are usually planned through interactions between medical engineers and surgeons. This interaction is time-consuming and can be interrupted by systematic or communication errors, causing this complex process to extend the postulated 7 days between diagnosis and the start of surgical therapy (13). Another potential disadvantage of the patient-specific reconstruction technique is the difficulty in adapting to situations in which the intraoperative surgical plan changes (e.g., positive margins on frozen section examination). Therefore, the time between surgical planning and surgery should also be minimized to avoid amplification of the tumor margins (14).

The technical aspects of producing patient-specific implants *via* selective laser melting and transport algorithms are relatively fixed and therefore cannot be accelerated significantly. To facilitate the production of patient-specific implants in less than one week, the focus must be turned to the planning process. To our knowledge, this is the first study on the influence of patient-specific implants on the preparation time of surgical tumor therapy.

Therefore, this study focused on the communication between engineers and surgeons and its immanent problems to improve the workflow in the planning process of patient-specific mandibular implants in a time-efficient manner. The investigators hypothesized that the level of training of engineers and complexity of planning would influence the duration of the overall process. The specific aims of this study were as follows: 1) to evaluate communication during the planning of patient-specific mandibular implants, 2) to identify possible measures of acceleration, and 3) to determine the effect of in-house training of engineers on planning speed.

## Materials and methods

This single-center, retrospective study included patients who were treated with a patient-specific mandibular implant (Individual Patient Solutions [IPS] Implants, KLS Martin Group, Tuttlingen, Germany) for continuous defects of the mandible from 2016 to 2021 at Hannover Medical School, Germany. The exclusion criteria were non-continuous defects and reconstructions requiring multiple implants, as communication in such cases was assumed to be more difficult and time-consuming regardless of the engineer’s training or implant complexity. Other exclusion criteria were missing data or a lack of consent for data usage. Mandibular reconstruction with patient-specific implants was planned using the IPS Gate platform (KLS Martin Group). The IPS Gate platform is a browser-based communication tool that uses a chat function and graphic interface for asynchronous planning of patient cases.

Some medical engineers using this platform were trained within the Department of Oral and Maxillofacial Surgery at Hannover Medical School, and therefore, attended surgery and gained insight into the surgeon’s needs. These medical engineers are categorized as “trained.” Other engineers were trained at the company without further exposure with the medical side of patient-specific implants. These medical engineers were categorized as “trained” or “untrained.”

**Figure 1:**
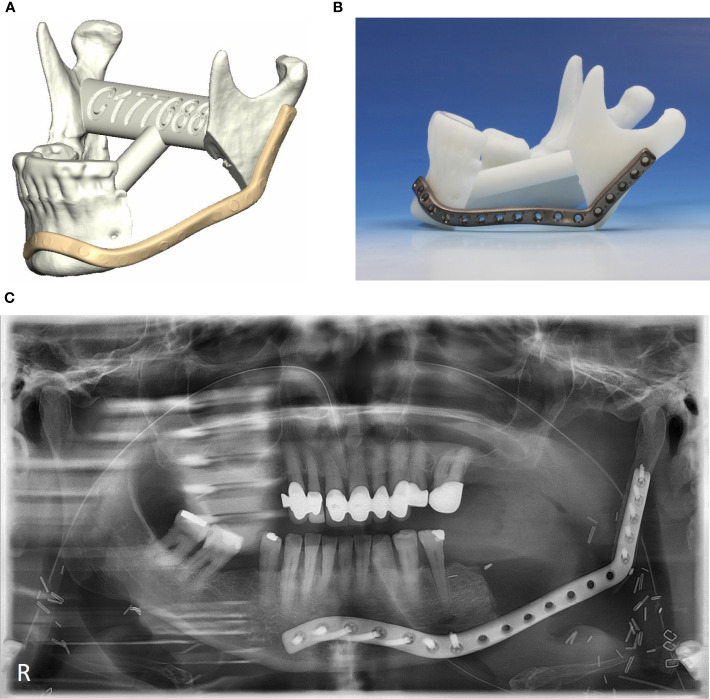
Non-complex patient-specific implants. **(A)** Digital planning, **(B)** patient-specific implant on plastic model, **(C)** postoperative orthopantomogram.

**Figure 2:**
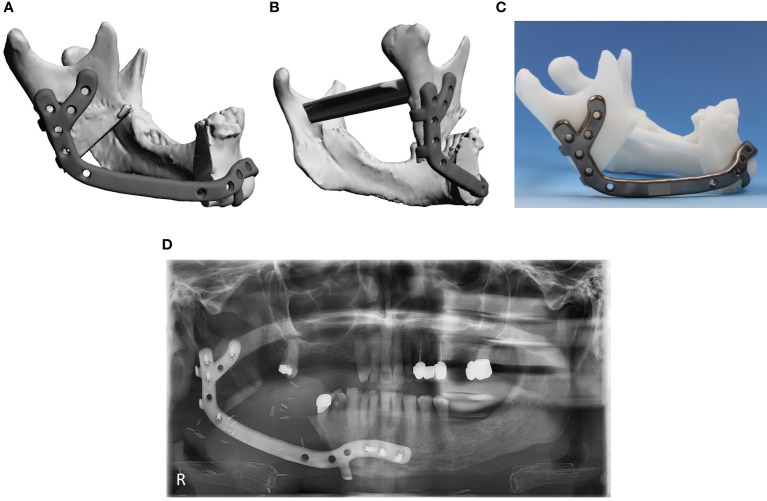
Complex patient-specific implant. **(A)** digital planning lateral view, **(B)** digital planning posterior-lateral view, **(C)** patient-specific implant on plastic model, **(D)** postoperative orthopantomogram.

Cases were also grouped as per the level of planning complexity. Patient-specific implants resembling conventional mandibular reconstruction plates were considered simple (**Figure 1**), whereas implants with a Y-shaped fixation at the mandibular ramus, implants reconstructing the chin area, and implants with additional retention hooks were considered complex (**Figure 2**).

### Variables

Training status (whether additional training was completed in the hospital or not) and implant complexity were regarded as predictor variables. The total duration of communication (time in days from the first to last message) was quantitatively recorded as the primary outcome variable. The secondary outcome variables were the need to postpone the planned operation, problems in the planning process, length of the messages (number of words per message), need for additional synchronous communication (yes/no), and need for changes to the original design (yes/no). As general patient information (age, sex) is irrelevant to engineers, these third category variables were not assessed in this study.

### Data collection

The chat logs saved on the IPS Gate platform were retrospectively evaluated. These include the total duration of communication, message length, and response time. The complexity of the implant was assessed based on standard triangle language files created during planning. Finally, the causes of communication problems were identified through qualitative evaluation.

### Data analysis

For group comparisons, the Mann–Whitney rank sum test was chosen because of the failure of the normality test (Shapiro-Wilk test). The chi-square test was used to compare categorical data. Statistical significance was set at P < 0.05, based on a 95% confidence interval. Statistical analyses were performed using Microsoft Excel 2016 (Microsoft Corporation, Redmond, WA, USA) and SigmaPlot 13.0 (Systat Software, Palo Alto, CA, USA).

### Ethics approval statement

This study was approved by the Institutional Ethics Committee of the investigators’ institution (reference number 9403_BO_K_2020) and was conducted in accordance with the Declaration of Helsinki. The participants provided written informed consent to participate in this study.

## Results

**Figure 3:**
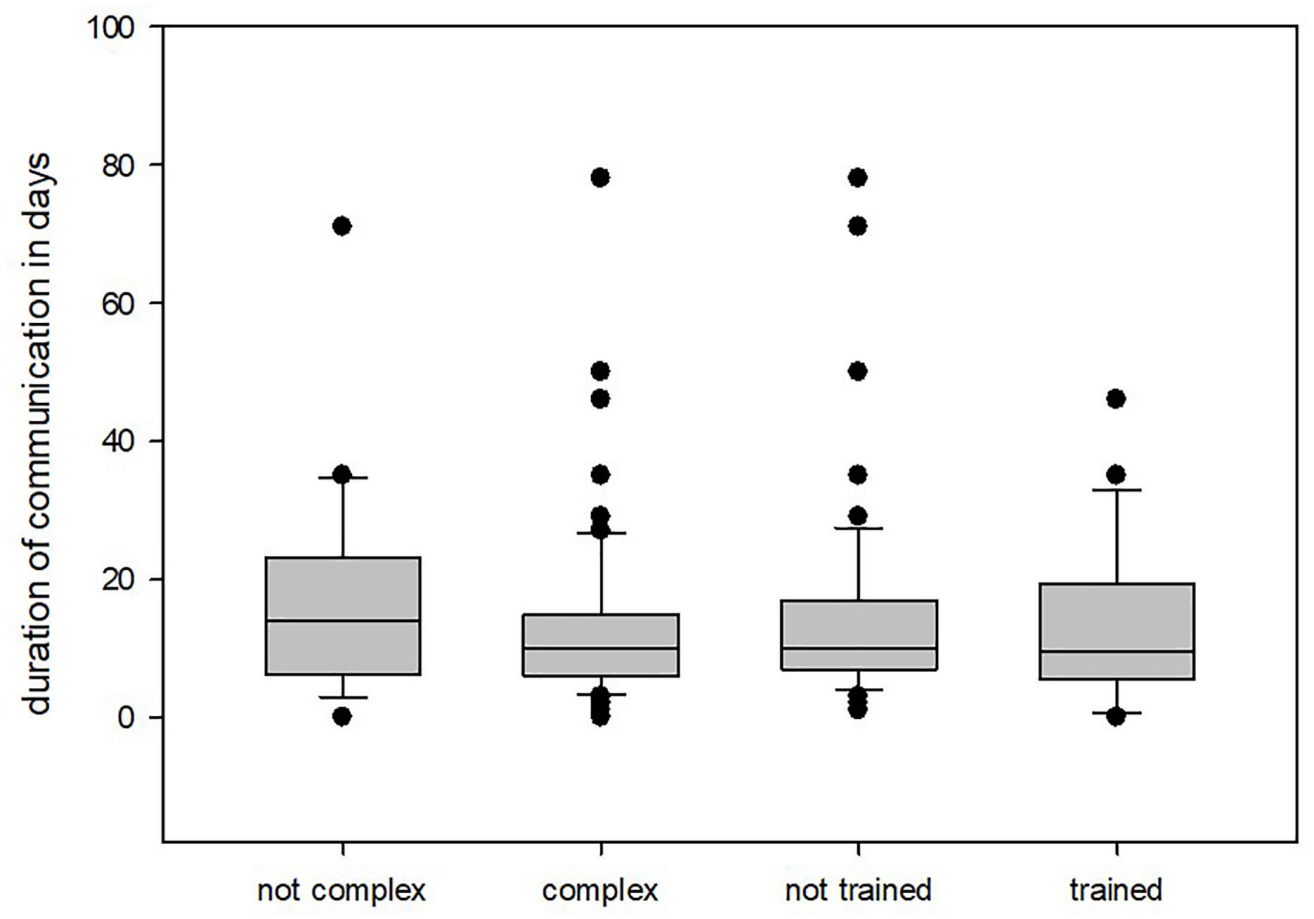
Duration of communication depending on engineer training or implant complexity.

A total of 83 patients were included in this study. The mean duration of communication was 14.05 ± 13.58 days. On average, 355.65 ± 251.61 words were written, with the engineers writing significantly more per message than the surgeons (200.23 ± 172.00 words vs. 155.42 ± 100.13 words; P = .001). The mean total duration of communication was not significantly shorter for simple patient-specific implants than for complex patient-specific implants (17.25 ± 15.77 days vs 12.97 ± 12.73 days; P = .337; **Figure 3**). For all cases, there was no statistically significant difference in the mean total duration of communication depending on the engineer’s training status (untrained 14.56 ± 14.51 days vs. trained 12.92. ± 11.21 days; P = .606; **Figure 3**). In 28 cases (33.73%), additional synchronous communication (web meetings or telephone calls) was required for clarification. There were no statistically significant differences in the need for additional synchronal communication depending on the engineers’ training status or the level of planning complexity (P = .0.700, P = .685; **Table 1**). However, a difference became evident when the need to postpone the planned operation was considered. In 16 of 83 cases (19.23%), the initial planned deadline for the operation could not be met. Of the 16 patients, 15 (93.75%) had complex patient-specific implants. Therefore, surgery for patients with a complex patient-specific implant had a significantly higher chance of being postponed than for those with simple patient-specific implants (P = 0.001; **Table 1**). However, the chance of postponing the operation was not significantly influenced by the training status of the engineer (P = 0.227; **Table 1**). The most frequent cause of communication problems was insufficient three-dimensional (3D) datasets (computed tomography [CT] or cone-beam CT [CBCT]). Specifically, either the slices were too thick or the relevant areas were not visible; such scans were unsuitable for implant planning (10.84%, n = 9). Cases planned based on CBCT were surprisingly less represented in this group than cases planned based on CT (two vs. six). Other causes were difficulty in making an appointment for synchronous communication (7.23%, n = 6) and changes in the engineer or surgeon involved (3.661%, n = 3). In almost three-quarters of the cases (72.29%, n = 60), the clinician requested changes to the initial plan. These requests were not significantly influenced by the complexity or training level of the planner (P = 0.16, P = 0.52, respectively; **Table 1**).

**Table 1:** Outcomes depending on implant complexity and engineer training status.

Implant design	Synchronous planning	Asynchronous planning	Design change	No design change	Postponed operation	Operation on time
Complex	22	14	48	15	15	48
Not complex	6	41	12	8	1	19
**P**	0.700^*^		0.524^*^		0.001^*^	
Engineer training status	Synchronous planning	Asynchronous planning	Design change	No design change	Postponed operation	Operation on time
Trained	8	18	20	6	3	23
Untrained	20	37	40	17	13	44
**P**	0.685^*^		0.524^*^		0.227^*^	

^*^chi-square test.

## Discussion

In this study, we investigated engineer-surgeon communication while planning patient-specific implants in malignant tumor cases and its impacts on the TTS.

Surprisingly, the duration of communication was not significantly influenced by predictor variables (additional training and implant complexity). Therefore, it can be concluded that the overall time to create patient-specific implants in HNSCC cases involving the mandible is not dependent on the design of the implant or training status of the involved engineer. Nevertheless, the average total planning time (approximately 14 days) was much longer than expected and far too long compared to the desired 7 days of surgical planning time. A closer look at the cases showed that a delay in the operation was necessary; in other words, the TTS was increased, and it became obvious that the complexity of the implant is not only an influence but also plays a crucial role. Of the 16 delayed cases, 15 involved complex patient-specific implants. Therefore, the risk for increased TTS was significantly linked to the complexity of the patient-specific implant (P = .001).

In another study performed by our group involving patient-specific orbital implants, we observed that in-house engineer training saved time during the planning process (13). Even if the present study does not support this claim, one conclusion can be drawn from both studies: implant complexity influences the planning process and TTS. Since the TTS is crucial for surgical treatment of malignancies, it is of utmost importance to avoid any unnecessary delay.

One possibility for speeding up the process of patient-specific implant creation without losing its benefits is the standardization of individualization. In other words, standardize all possible factors while maintaining patient-specific features. For example, keep the fixation areas to the bony defect margins patient-specific and follow standards concerning implant thickness and screw diameters. This standardization should include not only the implant itself, but also the planning process.

Yang et al. developed a surgeon-driven standard design process to optimize the planning process and concluded that the development of a surgeon-friendly software, preferably with an artificial intelligence algorithm, as well as the optimization of biomechanical properties and post-processing of 3D-printed surgical plates is necessary to standardize this fast-developing technology (15).

Other possibilities for optimizing the workflow in patient-specific treatment of malignancies would include the implementation of standards concerning imaging and 3D-data processing or deep learning algorithms (16). These standards or improvements should focus on our opinion on better software solutions using artificial intelligence and on the quality of 3D-imaging. The quality of 3D-imaging’s simple imaging parameters, such as the distance between two sectional views, seems to be more important than the type of imaging, such as CT or CBCT. This assumption is supported by the fact that, in our patient cohort, more image quality problems occurred in cases planned based on CT than on CBCT. To date, some companies still refuse to plant patient-specific implants based on CBCT scans without any scientific reasons.

Another possibility for speeding up the planning process is to simplify patient-specific implants. However, this would negate the benefits of these implants, such as the reconstruction accuracy (12, 17–20), and therefore, should not be considered.

This study had some limitations, mainly the retrospective nature of the evaluation. In addition, there could be confounding factors (e.g., holiday time) that influenced the communication duration, which we were unable to address. Furthermore, the sample size was small; therefore, a multicenter study may provide a more profound analysis of the influence of patient-specific implant creation on the TTS.

In conclusion, the process of patient-specific implant creation should be accelerated *via* standardization of the implant design and planning process. This can be achieved by using or developing modern software solutions for the planning process by addressing computer-aided design and communication pathways. In addition, the 3D-imaging quality plays an important role in the planning process and should, therefore, be predefined in coordination between surgeon and engineer to meet diagnostic and patient-specific treatment needs. If it is not possible to produce the patient-specific implant in a timely manner, it is often possible to change from a patient-specific treatment to a standard surgical procedure without a customized implant. Since TTS is a crucial factor in surgical tumor therapy that influences mortality, efforts should be made to keep it as low as possible.

## Data availability statement

The datasets presented in this article are not readily available because the raw data includes patient information and cannot be shared. Requests to access the datasets should be directed to SS, spalthoff.simon@mh-hannover.de.

## Author contributions

SS: Conceptualization, Methodology, Statistics, Writing – Original Draft. NN-R: Investigation, Methodology, Statistics, Writing – Original Draft. BR: Conceptualization, Methodology, Writing – Original Draft. PJ: Methodology, Writing – Review & Editing. FL: Writing – Review & Editing. N-CG and PK: Conceptualization, Project Administration, Writing – Review & Editing. All authors contributed to the article and approved the submitted version.

## Funding

This research did not receive any specific grant from funding agencies in the public, commercial, or not-for-profit sectors.

## Conflict of interest

The authors declare that the research was conducted in the absence of any commercial or financial relationships that could be construed as a potential conflict of interest.

## Publisher’s note

All claims expressed in this article are solely those of the authors and do not necessarily represent those of their affiliated organizations, or those of the publisher, the editors and the reviewers. Any product that may be evaluated in this article, or claim that may be made by its manufacturer, is not guaranteed or endorsed by the publisher.

## Abbreviations

3D, three-dimensional; CBCT, cone-beam computed tomography; CT, computed tomography; HNSCC, head and neck squamous cell carcinoma; IPS, Individual Patient Solutions; TTS, time to surgery.
